# Survival, safety, and prognostic factors for outcome with Regorafenib in patients with metastatic colorectal cancer refractory to standard therapies: results from a multicenter study (REBACCA) nested within a compassionate use program

**DOI:** 10.1186/s12885-016-2440-9

**Published:** 2016-07-07

**Authors:** Antoine Adenis, Christelle de la Fouchardiere, Bernard Paule, Pascal Burtin, David Tougeron, Jennifer Wallet, Louis-Marie Dourthe, Pierre-Luc Etienne, Laurent Mineur, Stéphanie Clisant, Jean-Marc Phelip, Andrew Kramar, Thierry Andre

**Affiliations:** Medical Oncology, Centre Oscar Lambret and Catholic University, Lille, France; Medical Oncology, Centre Léon Bérard, Lyon, France; Medical Oncology, Paul Brousse University Hospital, Villejuif, France; Medical Oncology, Gustave Roussy, Villejuif, France; Gastroenterology, University Hospital, Poitiers, France; Methodology and Biostatistics, Centre Oscar Lambret, Lille, France; Medical Oncology, Clinique Sainte Anne, Strasbourg, France; Medical Oncology, Hopital Privé des Côtes d’Armor, Plérin, France; Radiation and Medical Oncology, Institut Sainte-Catherine, Avignon, France; Clinical Research Unit, Centre Oscar Lambret, Lille, France; Gastroenterology, University Hospital, Saint Etienne, France; Medical Oncology, Saint Antoine Hospital, and University Pierre et Marie Curie (UMPC), Paris VI, Paris, France; Department of Medical Oncology, Centre Oscar Lambret, 3, rue F Combemale, 59000 Lille, France

**Keywords:** Colorectal cancer, Metastatic disease, Regorafenib, Antiangiogenic, Cohort study

## Abstract

**Background:**

Randomized trials have shown a survival benefit for regorafenib over placebo in patients with metastatic colorectal cancer (mCRC) that progressed after standard therapies. We evaluated survival and safety outcomes in patients treated with regorafenib in a real-life setting.

**Methods:**

REBECCA is a cohort study nested within a compassionate use program designed to evaluate survival, safety, and potential prognostic factors for outcome associated with regorafenib in patients with mCRC refractory to standard therapies. Treatment effects according to various patient and tumour characteristics were evaluated using univariate and multivariate Cox proportional hazards regression models.

**Results:**

Of 1178 patients in the compassionate use program, 654 were in the full analysis set. Median follow-up was 16.5 months. Median survival was 5.6 months. The 12-month survival rate was 22 %. Survival was independently and unfavourably affected by the following variables: poor performance status, short time from initial diagnosis of metastases to the start of regorafenib, low initial regorafenib dose, >3 metastatic sites, presence of liver metastases, and *KRAS* mutations. We identified prognostic groups of patients with low, intermediate, and high risk of death, with a median survival of 9.2, 5.2, and 2.5 months, respectively. Five-hundred-twenty-four patients (80 %) experienced at least one regorafenib-related adverse event, most commonly, fatigue, hand-foot skin reaction, diarrhea, anorexia, arterial hypertension, and mucositis.

**Conclusion:**

The safety and efficacy profile of regorafenib in REBECCA are similar to those in randomized trials. Our prognostic model identified subgroups of mCRC patients who derived a minimal and maximum benefit from regorafenib.

**Trial registration:**

Clinicaltrials.gov NCT02310477.

## Background

Regorafenib is an oral multikinase agent that inhibits angiogenic and stromal receptor tyrosine kinases such as VEGFR1/3, PDGFR-b, FGFR-1, and TIE-2. Together with inhibition of oncogenic receptor tyrosine kinases c-KIT, and RET, regorafenib also blocks the activity of signaling kinases such as RAF1 and B-RAF [1].

Two phase III trials demonstrated a significant overall survival (OS) benefit for regorafenib over placebo in patients with metastatic colorectal cancer (mCRC) who progressed on standard therapies [2, 3]. As a result, regorafenib was granted marketing authorization in many countries for mCRC at a dose of 160 mg/day for the first 3 weeks of each 4-week cycle.

Because patient selection and real-life prescribing conditions may differ from those of randomized clinical trials, we evaluated the efficacy, safety, and potential predictors of outcome in patients treated with regorafenib in this setting. Once approved in France for the treatment of mCRC, but before full reimbursement was possible, the French authorities made regorafenib available through a *Temporary Use Authorization* (Autorisation Temporaire d’Utilisation or ATU) program. This is an exceptional procedure approved by the French National Agency for Medicines and Health Products Safety (ANSM) intended to provide early access to new medicines, especially for unmet needs. Prescribing conditions, based on the ATU label, are less stringent than selection criteria in clinical trials. The ATU data contributes to better understanding of the medicinal product and a reliable evaluation of its benefit/risk ratio in the real-life setting.

The REgorafeniB in mEtastatic Colorectal cancer: a French Compassionate progrAm (REBECCA) is a cohort study nested within the ATU designed to evaluate the efficacy and safety of regorafenib in real-life clinical practice for mCRC patients who have been previously treated with or are not considered candidates for standard therapies.

## Methods

**Fig. 1:**
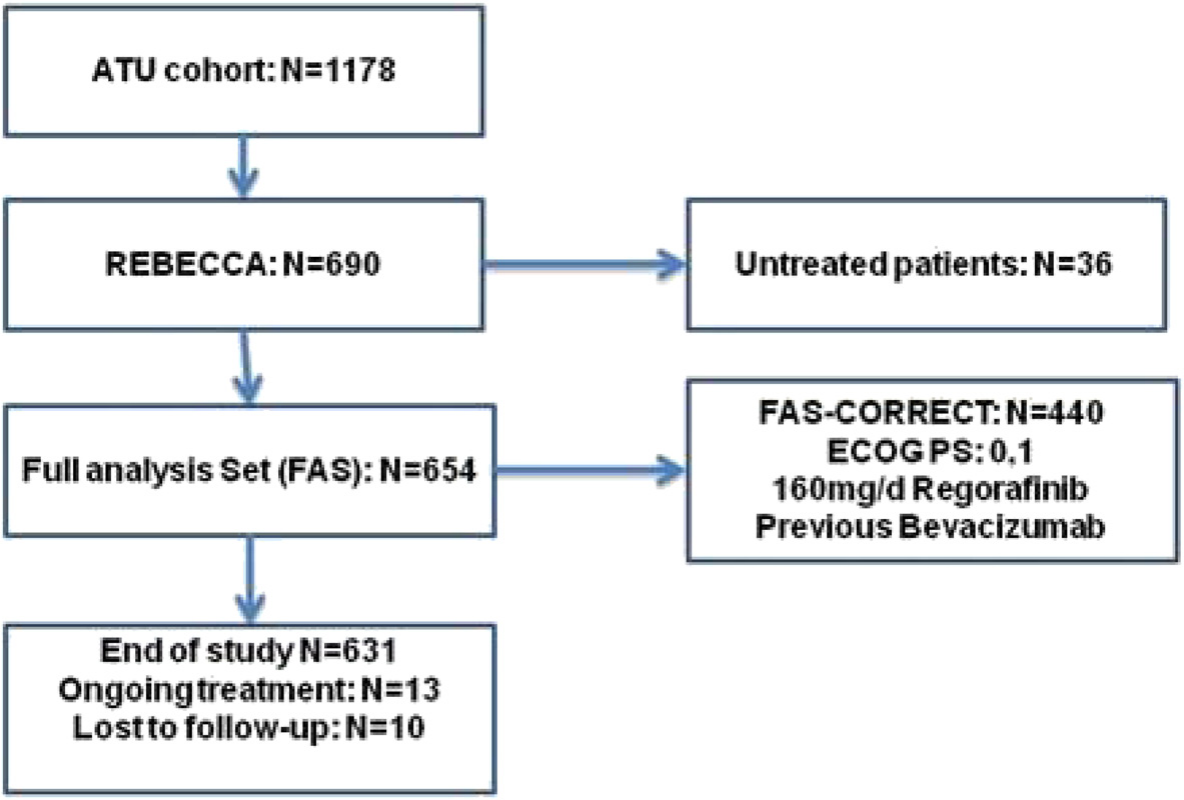
REBECCA flow chart

This study was performed according to the Declaration of Helsinki. Written consent was not required from patients, according to French laws governing noninterventional studies. However, patients alive at the time of the study received full information by the investigators, regarding the research and the anonymous data collection. If a patient refused participation, the registration was not performed. Ethic approvals were obtained by submission of the study to the “Consultative Committee for Data processing in Research in the Health field” (CCTIRS, file 14–042, approval date: January 15th, 2014), and to “National Committee of data processing for data protection” (CNIL, file 914071, approval date: May 22nd, 2014). The study is registered in clinicaltrials.gov (NCT02310477). REBECCA was conducted in France at 136 institutions that completed an updated case report form, including 42 university/comprehensive cancer hospitals, 45 general hospitals, and 39 private practice clinics. Because some physicians or patients did not agree to participate to that study, the study population was a subset of the 1178 adults with histologically proven mCRC satisfying the criteria for regorafenib treatment validated by the ANSM in the ATU (Fig. 1). Data were collected from 690 patients and the 654 who received at least one regorafenib dose comprised the Full Analysis Set (FAS). Case report forms from at least 60 % of patients were randomly monitored for accuracy. Patients were treated with regorafenib from October 2012 to January 2014. The median follow-up was 16.5 months (range: 1 day–21.9 months). Data cutoff was December 16, 2014. Baseline demographic and clinical variables were retrospectively collected, whereas survival data and post progression treatments were prospectively collected. Baseline demographic and clinical variables included: age, sex, BMI, ECOG PS, institution type, number of treated patients per center, primary tumour location, time from initial diagnosis of metastases and start of regorafenib, synchronous/metachronous metastases, number of metastatic sites, sites of metastases, *KRAS* mutational status, previous bevacizumab therapy, time from last bevacizumab, and initial regorafenib dose. Treatment compliance, dose-intensity, adverse events (AEs), pre- and post-regorafenib treatments, and potential prognostic factors for OS were also evaluated. Severity of AEs was graded using National Cancer Institute Common Terminology Criteria for Adverse Events, version 4.0.

### Statistics

Descriptive statistics were used to summarize data. Median follow-up was calculated by the inverse Kaplan-Meier method. All time-to-event variables were calculated from the date of first regorafenib administration. OS was calculated to the date of death; patients alive at the time of analysis were censored at the last observation. Progression-free survival (PFS) was defined as the time to first progression or death, whichever came first; non-progressing patients alive at the time of analysis were censored at last follow-up. Hazard ratios (HR) were estimated from the semi-parametric Cox proportional hazards model. Treatment effects were evaluated by univariate and multivariate Cox proportional hazards regression models for OS.

For prognostic scores, we first did a univariate analysis of demographic and clinical variables and retained those significant at the 0.10 level in the multivariate model. Next, we combined significant prognostic factors from the multivariate model to define a prognostic score for patients with similar risks of death by rounding regression coefficients to the nearest integer to obtain relative weights of the variables and summing them to obtain a total score. This score ranged from 0 to 10 and was then reduced to three prognostic groups by combining adjacent non-significant categories (Wald test) from hierarchically defined dummy indicator variables to obtain three categories of patients with significantly different risks of death. Other potential prognostic variables were then added to this reduced model and tested for significance using the likelihood ratio statistic.

Exploratory *post hoc* analyses assessed the OS prognostic score in a subgroup of FAS patients having characteristics similar to patients in CORRECT [2], including ECOG PS 0–1, 160 mg initial regorafenib dose, and previous exposure to bevacizumab (FAS-CORRECT population, *n* = 440). We also evaluated the impact of toxicity type on OS, recalibrated from 1 month after the start of regorafenib.

## Results

Baseline characteristics are listed in Table 1. Thirty-one percent and 48 % of patients had 1 and 2 or more metastatic sites, respectively, mostly to liver and lung. Thirty-five percent of patients had at least 3 prior lines of treatment for metastatic disease and 15 % had 5 lines or more. Almost all received prior oxaliplatin and irinotecan (99 %), 92 % had prior bevacizumab, and 283/291 (97 %) of patients with *KRAS* wild-type tumours had previously received anti-EGFR therapy. The median time since last bevacizumab was 4.4 months. Baseline patient and disease characteristics were similar to those of the overall ATU population (data not shown). Patients were similarly distributed among university/comprehensive cancer hospitals, general hospitals, and private clinics. A majority of centres (55 %) included 7 patients or more.

**Table 1:** Selected baseline patient and disease characteristics

	FAS population
Age (years): median (range)	64 (25–91)
Age (years): *n* (%)	
< 65	343 (52.4)
≥ 65	311 (47.6)
BMI (Kg/m^2^)	24 (14.1–49.1)
ECOG Performance Status: *n* (%)	
0	200 (30.7)
1	383 (58.7)
2	60 (9.2)
3	9 (1.4)
Missing	2
Site of Primary tumour: *n* (%)	
Colon	445 (69.9)
Rectum	186 (29.9)
Colon and rectum	5 (0.2)
Missing	18
Timing of metastases: *n* (%)	
Synchronous	416 (65.6)
Metachronous	218 (34.4)
missing	20
Delay from initial diagnosis of metastases (months): median (range)	31 (0.8–156.5)
Delay from initial diagnosis of metastases (months): *n* (%)	
< 18	134 (20.6)
≥ 18	518 (79.4)
Missing	2
KRAS mutational status: *n* (%)	
Wild	291 (46.8)
Mutated	331 (53.2)
Missing	32
Initial dose of REG, mg/d, 3 w/4: *n* (%)	
40	3 (0.5)
80	39 (6)
120	89 (13.6)
160	522 (79.9)
Missing	1

Median regorafenib treatment duration was 2.2 months (range, 0.1–20.5) and 13 patients were still on therapy at data cut-off. An initial 160 mg daily dose was given to 80 % of patients (Table 1). The actual mean dose-intensity during the first month of regorafenib was 751.2 mg per week (SD+/−142.2), i.e. 89 % of the expected figure. Fifty percent of patients had a treatment modification (dose reduction or interruption). A total of 204 patients (31 %) either temporarily or permanently stopped regorafenib before progression, mainly for toxicity (Table 2) and 43 % had dose reductions mainly for toxicity or general health status deterioration. Median time to first treatment modification was 0.7 months (range, 0.03–6.01). Of patients starting cycles 3 and 4, 50 and 39 % received the full regorafenib dose, respectively.

**Table 2:** Treatment modifications

	FAS population
Dose interruptions^a^: *n* (%)	204 (31.2)
Reasons: *n* (%)	
Patient willingness	8 (3.9)
General health deterioration	29 (14.2)
Adverse event (treatment-related or not)	173 (84.8)
Other	9 (4.4)
Dose reduction: *n* (%)	278 (42.5)
Reasons: *n* (%)	
Patient willingness	6 (2.2)
General health deterioration	66 (23.7)
Disease progression	2 (0.7)
Adverse event (treatment-related or not)	226 (81.3)
Other	20 (7.2)
Any kind of dose modification: *n* (%)	329 (50.3)

^a^Permanent or temporary interruption

Median OS was 5.6 months (interquartile range [IQR], 2.4–11.4). Twelve-month OS rate was 22 %. OS was worse in patients with low BMI, ECOG PS >0, and in patients managed in university/comprehensive cancer centers or general hospitals. A short time since the diagnosis of metastases, the presence of synchronous or liver metastases, a high number of metastatic sites, a low initial regorafenib dose, a short time since prior bevacizumab, and *KRAS* mutations were also associated with a shorter OS. Dose-intensity at 1 or 2 months after starting regorafenib did not impact OS. Multivariate analysis showed OS was independently affected by ECOG PS, time since initial diagnosis, initial daily dose, number of metastatic sites, liver metastases, and *KRAS* mutation (Table 3).

**Table 3:** OS multivariate analysis on FAS and FAS CORRECT populations

Overall survival	Hazard ratio (95 % CI)	*P*	Relative weight
FAS Population			
ECOG Performance Status		<0.001	
0	1		0
1	1.54 (1.26–1.88)		+2
≥ 2	3.43 (2.50–4.70)		+4
Time since initial diagnosis		<0.001	
≥ 18 months	1		0
< 18 months	1.72 (1.40–2.13)		+2
Initial daily dose of regorafenib		0.042	
160	1		0
< 160	1.26 (1.01–1.57)		+1
Number of metastatic sites		0.020	
< 3	1		0
3+	1.29 (1.04–1.60)		+1
Liver metastases		<0.001	
No	1		0
Yes	1.61 (1.29–2.01)		+2
KRAS		0.016	
Wild-type	1		0
Mutated	1.25 (1.04–1.49)		+1
FAS CORRECT Population			
ECOG Performance Status		0.001	
0	1		0
1	1.45 (1.16–1.81)		+1
≥ 2			
Time since initial diagnosis		0.001	
≥ 18 months	1		0
< 18 months	1.55 (1.21–1.99)		+1
Number of metastatic sites		0.013	
< 3	1		0
3+	1.38 (1.07–1.77)		+1
Liver metastases		<0.001	
No	1		0
Yes	1.65 (1.28–2.13)		+1

**Fig. 2:**
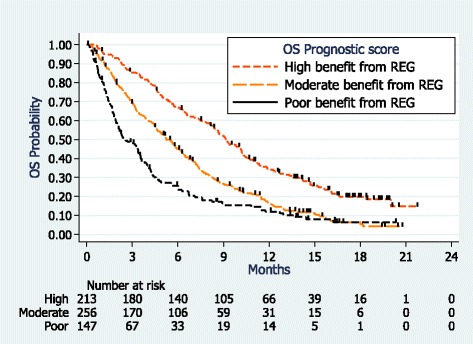
OS according to prognostic scores (FAS population)

The low-risk group (regorafenib high OS benefit) with a maximum prognostic score of 3, represented 34 % of patients having a median OS of 9.2 months (Table 4, Fig. 2). The moderate-risk group score was 4 or 5 and represented 42 % of patients with a median OS of 5.2 months. The high-risk group (low OS benefit) had a score of 6 or more and represented 24 % of patients having a median OS of 2.5 months.

**Table 4:** OS prognostic score in the FAS and the FAS CORRECT populations

FAS Population *n* = 654		Median OS	6-month OS rate
Score equal to 0,1, 2, or 3 (high benefit from REG, *n* = 213)	**1**	**9.2**	**67 %**
Score equal to 4 or 5 (moderate benefit from REG, *n* = 256)	1.78 (1.45–2.18)	5.2	45 %
Score equal to 6+ (low benefit from REG, *n* = 147)	2.70 (2.13–3.42)	2.5	26 %
FAS-CORRECT Population *n* =440			
Score equal to 0 or 1 (high benefit from REG, *n* = 185)	1	8.7	64 %
Score equal to 2 (moderate benefit from REG, *n* = 206)	1.67 (1.34–2.09)	5.5	46 %
Score equal to 3 (low benefit from REG, *n* = 48)	2.54 (1.79–3.60)	3.4	35 %

Relative weights of the variables found independently related to survival (see Table 3) were added to obtain a total score. This score ranged from 0 to 10, and was then reduced to obtain three categories of patients with significantly different risks of death

A *post hoc* exploratory analysis of the FAS-CORRECT population showed a median OS of 6.3 months and a 12-month OS rate of 23 %. From the variables found significantly related to OS after univariate and multivariate analyses (data not shown), the OS prognostic score of the FAS-CORRECT population was similar to that of the FAS (Table 4). However, fewer FAS-CORRECT patients (11 vs 24 %) were classified as low OS benefit from regorafenib and more were classified as high OS benefit (42 vs 34 %).

The median time to first tumour assessment from treatment initiation was 2.6 months (range, 0.3–6.3). Median PFS was 2.7 months (IQR, 1.6–4.6) and 12-month PFS rate was 7 %.

### Safety

Overall 524/654 (80 %) of patients had at least one AE the investigator considered regorafenib-related (Table 5). Worst AE grade was not available for 12 patients. Rates of worst-grade AEs in 512 patients were: grade 1 (*n* = 53, 10 %), grade 2 (*n* = 171, 33 %), grade 3 (*n* = 231, 45 %), grade 4 (*n* = 55, 11 %) and grade 5 (*n* = 2, <1 %). Two deaths (one fronto-parietal lobar cerebral hematoma and one bowel perforation) were suspected to be related to regorafenib. Most frequently (>10 %) reported regorafenib-related AEs were fatigue, hand-foot skin reaction (HFSR), diarrhea, anorexia, arterial hypertension, and mucositis (Table 5). Most common grade 3–4 regorafenib-related AEs were fatigue and HFSR. Occurrence of HFSR during the first month of treatment was associated with a statistically significantly better OS (HR, 0.61 [95 % CI, 0.50–0.74]; *p* < 0.0001). Patients (*n* = 197) who presented with HFSR during the first month of treatment had apparently better median survival (7.7 months vs 4.1 months), and 6-months survival rate (61 vs 39 %), that patients who did not (*n* = 392). This was not observed for other toxicities.

**Table 5:** Safety (most frequent AEs related to REG clinical use) (FAS population, *n* = 654)

Adverse events	Any Grade *N* (%)	Grade 3–4 *N* (%)
Any event	524 (80.1)	286 (43.7)
Fatigue	271 (41.4)	95 (14.5)
Hand and Food Skin Reactions	189 (28.9)	59 (9)
Diarrhea	123 (18.8)	28 (4.3)
Anorexia	96 (14.7)	19 (2.9)
Hypertension	72 (11)	30 (4.6)
Mucositis	72 (11)	8 (1.2)
Weight loss	33 (5)	4 (<1)
Rash or desquamation	26 (4)	8 (1.2)
Thrombopenia	21 (3.2)	1 (<1)
Muscle pain	9 (1.4)	1 (<1)
Proteinuria	8 (1.2)	2 (<1)
Hyperbilirubinemia	7 (1.1)	0

A total of 223 patients (34 %) received at least one post-regorafenib therapy, including 89/223 patients who received more than one line of post-regorafenib treatment. 108/223, 100/223, and 15/223 patients were treated with chemotherapy only, with chemotherapy plus targeted therapies, and with targeted therapies only, respectively. Overall, a rechallenge with anti-EGFR agents was observed in 52 patients with presumably *KRAS* wild-type tumours. The median OS (from date of progression on regorafenib to death) in 152 patients who received post-progression treatment was 7.9 months (range, 6.1–9.0), and was 3.4 months (range, 3.1–3.8) in 431 patients who did not receive any post-progression therapy.

## Discussion

The CORRECT trial showed that regorafenib significantly improves OS (HR [95 % CI]: 0.77 [0.64–0.94]) and PFS (0.49 [0.42–0.58]) versus placebo in patients with mCRC that progressed after standard therapies [2]. These results were confirmed, although with a greater survival benefit for regorafenib, by a recently published trial of Asian patients, a significant proportion of whom had not received a prior targeted agent [3]. In these trials, a vast majority of regorafenib-treated patients had at least one AE, most commonly HFSR, fatigue, diarrhea, hypertension, anorexia, oral mucositis, hoarseness, liver enzymes, and bilirubin changes. One third to half of these AEs were classified as grade >2, with fatigue and HFSR (no grade 4) being the most frequent [2, 3].

Because patients and prescribing conditions in real-life may differ from phase III trials, data on efficacy, safety, and on potential predictors of outcomes in patients treated with regorafenib in this setting are important for future clinical use and to potentially identify patients who may benefit most from treatment.

Indeed, REBECCA should be interpreted carefully because its limitations due to its retrospective design and its exploratory subgroup analyses. However, it provides insights into real-life clinical practice in a large cohort of 654 carefully monitored patients who represent the parent ATU population.

As prescribing conditions in the ATU are less stringent than in clinical trials, it is not surprising that some patient characteristics in REBECCA differed from those in CORRECT [2]. For example, in REBECCA 11 % of patients had a baseline ECOG PS >1 and 20 % initially received <160 mg/day of regorafenib, whereas in CORRECT all patients were ECOG PS0–1 at baseline and all started at 160 mg/day. Median OS in REBECCA was 5.6 months and 22 % of patients were alive 12 months after starting regorafenib. Although consistent with CORRECT [2] (median OS 6.4 months, 12-month survival 24 %), the slightly lower median OS may be related to different patient characteristics in the real-life setting. Limiting our analysis to REBECCA patients having similar baseline characteristics as patients in CORRECT (FAS-CORRECT), showed results closer to the clinical trial (median OS 6.3 months; 12-month OS rate 22.8 %). Median PFS in REBECCA (2.9 months) appeared better than in CORRECT (1.9 months), but this should be interpreted with caution because the median time to first tumor assessment was longer in real-life (13 weeks) than in the prospective trial (8 weeks).

No predictive factors for OS have been identified for mCRC patients treated with regorafenib, although a subgroup analysis from CONCUR suggests that prior exposure to targeted therapies (bevacizumab and/or anti-EGFR) may influence OS [3]. In CORRECT, no association between *KRAS*, *BRAF*, and *PIK3CA* mutation status and outcomes with regorafenib was identified [4]. Conversely, in our real-life study we identified several variables independently associated with OS. High ECOG PS, a shorter time from initial diagnosis of metastases, an initial REG dose <160 mg, >3 metastatic sites, liver metastases, and *KRAS* mutations, were independently associated with poorer survival, suggesting that with these easily-collected baseline variables, patients could be classified into similar prognostic groups. More importantly, we were able to identify patients having a small probability of benefiting from regorafenib. This predictive pattern was reproduced in the FAS-CORRECT population that was clinically similar to CORRECT, in which we identified patients (about 10 % of the FAS population) with a particularly poor outcome (median OS = 3.4 months).

The AEs reported in REBECCA are consistent with previous studies of regorafenib in mCRC, with fatigue, HFSR, hypertension, and diarrhea being the most frequent. However, it appeared than the rate of treatment-related AEs of any grade is lower in REBECCA (80 %), than that in CORRECT and CONCUR (97 and 93 %, respectively). This discrepancy may be related to the retrospective design of our study.

Additionally, we showed that the occurrence of HFSR within the first month of treatment was related to a better OS. Obviously, this finding needs to be confirmed, as it was based on an unplanned exploratory analysis using a landmark method restricted to patients who were alive without progression at 1 month. Whether or not the occurrence of a cutaneous AE within the first month of regorafenib treatment is predictive for outcome has to be confirmed prospectively by a multivariate analysis of potential predictive markers.

Forty-three percent of the patients had a dose reduction for toxicity or general health status deterioration. Of patients starting the fourth cycle, only 39 % received regorafenib at full dose. A similar proportion of dose reductions were also found in CORRECT [2] and in CONSIGN, a large (*n* = 2872) open-label phase IIIb study of patients with mCRC presented at the 2015 World Congress on Gastrointestinal Cancer [5]. Obviously, the question of regorafenib dose is critical, as it is related to efficacy, safety, compliance, regulatory issues, and medication costs. At least one randomised trial comparing a lower dose of regorafenib with a standard dose in patients with refractory mCRC is underway (NCT02368886).

Finally, one-third of patients who were treated after progression on regorafenib had a clinically better OS which, as has been suggested, shows that despite unproven efficacy, there may be room for further treatment in selected patients [6].

## Conclusion

The survival and safety profiles of regorafenib in the real-life setting are similar to those reported in prospective trials. We found that some variables may be associated with either better or worse OS and that our prognostic model was able to categorize mCRC patients into groups deriving a minimal and maximum benefit from regorafenib. Whether our findings are of predictive, and/or of prognostic value is not clear and warrants further investigation.
