# Erratum to: Survival, safety, and prognostic factors for outcome with Regorafenib in patients with metastatic colorectal cancer refractory to standard therapies: results from a multicenter study (REBECCA) nested within a compassionate use program

**DOI:** 10.1186/s12885-016-2559-8

**Published:** 2016-07-25

**Authors:** Antoine Adenis, Christelle de la Fouchardiere, Bernard Paule, Pascal Burtin, David Tougeron, Jennifer Wallet, Louis-Marie Dourthe, Pierre-Luc Etienne, Laurent Mineur, Stéphanie Clisant, Jean-Marc Phelip, Andrew Kramar, Thierry Andre

**Affiliations:** Medical Oncology, Centre Oscar Lambret and Catholic University, Lille, France; Medical Oncology, Centre Léon Bérard, Lyon, France; Medical Oncology, Paul Brousse University Hospital, Villejuif, France; Medical Oncology, Gustave Roussy, Villejuif, France; Gastroenterology, University Hospital, Poitiers, France; Methodology and Biostatistics, Centre Oscar Lambret, Lille, France; Medical Oncology, Clinique Sainte Anne, Strasbourg, France; Medical Oncology, Hopital Privé des Côtes d’Armor, Plérin, France; Radiation and Medical Oncology, Institut Sainte-Catherine, Avignon, France; Clinical Research Unit, Centre Oscar Lambret, Lille, France; Gastroenterology, University Hospital, Saint Etienne, France; Medical Oncology, Saint Antoine Hospital, and University Pierre et Marie Curie (UMPC), Paris VI, Paris, France; Department of Medical Oncology, Centre Oscar Lambret, 3, rue F Combemale, 59000 Lille, France

## Erratum

After publication of the original article [1], an error was noticed within the title: acronym “REBECCA” was erroneously written as “REBACCA”. The original article has now been updated with the correct title which can also be found above. The publisher would like to apologise for this error and for any inconvenience this may have caused.
